# Spondyloarthritis Research Consortium of Canada sacroiliac joint inflammation and structural scores: change score reliability and recalibration utility in children

**DOI:** 10.1186/s13075-020-02157-4

**Published:** 2020-03-24

**Authors:** Pamela F. Weiss, Walter P. Maksymowych, Rui Xiao, David M. Biko, Michael L. Francavilla, Robert G. Lambert, Jacob L. Jaremko, Merav Heshin-Bekenstein, Timothy G. Brandon, Nancy A. Chauvin

**Affiliations:** 1 Division of Rheumatology at the Children’s Hospital of Philadelphia and Center for Clinical Epidemiology and Biostatistics, Department of Pediatrics, Perelman School of Medicine at the University of Pennsylvania, Philadelphia, USA; 2 The Children’s Hospital of Philadelphia, Roberts Center for Pediatric Research, 2716 South Street, Room 11121, Philadelphia, PA 19104 USA; 3 Department of Medicine at the University of Alberta and CaRE Arthritis, Edmonton, Canada; 4 Department of Biostatistics, Epidemiology and Informatics, Perelman School of Medicine at the University of Pennsylvania, Philadelphia, USA; 5 Department of Radiology at the Children’s Hospital of Philadelphia and Department of Radiology, Perelman School of Medicine at the University of Pennsylvania, Philadelphia, USA; 6 Department of Radiology and Diagnostic Imaging at the University of Alberta, Edmonton, Canada; 7 Department of Pediatrics, Division of Rheumatology, University of California San Francisco and Dana Children’s Hospital of Tel Aviv Medical Center, Tel Aviv University, Tel Aviv, Israel; 8 Division of Rheumatology and Center for Pediatric Clinical Effectiveness at the Children’s Hospital of Philadelphia, Department of Pediatrics, Philadelphia, USA; 9 Department of Radiology at Penn State Health Milton S. Hershey Children’s Hospital, Hershey, PA USA

**Keywords:** Sacroiliac joint, Magnetic resonance imaging, Pediatrics, Spondyloarthritis, Calibration, Disease progression

## Abstract

**Background:**

The SPARCC sacroiliac joint inflammation (SIS) and structural (SSS) scores are reliable measures to quantify abnormalities in the pediatric sacroiliac joint. We aimed to evaluate the utility of online calibration modules for the SIS and SSS and the reliability of their component change scores.

**Methods:**

Change score reliability of 6 raters was assessed by overall and pairwise intraclass correlation coefficients (ICCs) before and after the use of real-time iterative calibration (RETIC) modules for both the SIS and SSS comprised of 20 adult cases. Acceptable ICC for change scores was > 0.7 for SIS and > 0.5 for all SSS components. Sensitivity to change was assessed by the standardized response mean (SRM).

**Results:**

In scoring exercise 1, the SIS had acceptable reliability with a change score ICC of 0.80 and sclerosis was the only SSS lesion that met the acceptability threshold with a change score ICC of 0.52. After RETIC calibration, the SIS overall (ICC = 0.83) and mean pairwise (ICC = 0.83) change scores remained reliable with a large SRM (0.90). All SSS components except sclerosis met the overall and mean pairwise change score ICC acceptability thresholds—backfill: overall = 0.54, mean pairwise = 0.50; fat metaplasia: overall = 0.65, mean pairwise = 0.57; erosion: overall = 0.60, mean pairwise = 0.58; and ankylosis: overall = 0.96, mean pairwise = 0.96. The SSS RETIC module augmented the number of SSS components surpassing the acceptability threshold from 1 to 4. Sensitivity to change, as measured by the SRM, was large for erosion (0.96), moderate for backfill (0.55) and sclerosis (0.70), and small for fat metaplasia (0.36) and ankylosis (0.28).

**Conclusion:**

RETIC modules improved the overall reliability of SPARCC SIS and SSS change scores for previously calibrated raters. SIS recalibration was not as helpful to the most experienced raters who achieved high levels of agreement before recalibration. The SPARCC SIS and all SSS components except sclerosis are reliable measures to quantify change over time in children. A pediatric-specific RETIC tool should be developed to enhance the calibration of readers.

## Background

Magnetic resonance imaging (MRI) is increasingly relied upon for detection of early inflammatory changes in the sacroiliac joint. Most pediatric centers globally evaluate the presence or absence of bone marrow edema, erosion, and sclerosis. However, there is an unmet need for objective and reliable MRI tools to quantitatively assess inflammation and structural lesions in the sacroiliac joints of children with spondyloarthritis. Without tools to objectively measure the severity of disease in the pediatric sacroiliac joint, quantitative assessment of interval change and response to both existing biologics and emerging targeted drugs for axial arthritis is not possible.

The Spondyloarthritis Research Consortium of Canada (SPARCC) sacroiliac joint (SIJ) inflammation (SIS) and structural scores (SSS) were developed and validated for use in adults with axial spondyloarthritis [1–3]. The SIS [1] evaluates the presence, depth, and intensity of bone marrow inflammation while the SSS [2] assesses a spectrum of structural lesions of the sacroiliac joint on MRI including backfill, fat metaplasia, erosion, and ankylosis; for pediatric cases, we also included sclerosis [4]. Scoring for the SIS and SSS is assessed dichotomously as the presence/absence of each lesion in SIJ quadrants or halves on consecutive slices through the cartilaginous part of the joint using semicoronal views. The minimally important change in disease is established for these tools [5] in adults and both have been successfully leveraged for clinical trials [6, 7].

The SPARCC SIS and SSS have face validity and reliability for the assessment of the pediatric joint based on cross-sectional data [4, 8, 9]. One recent pilot study demonstrated high rater agreement in SIS change scores for a pair of radiologist raters assessing 15 juvenile spondyloarthritis subjects with two or more scans [9]. Due to patterns and variations of normal development [10], scoring of pediatric SIJ pathology is particularly challenging. Even raters experienced in scoring adult SIJ may benefit from enhanced calibration to optimize inter-reader reliability. Real-time iterative calibration (RETIC) with real-time feedback is a new concept in imaging research based on principles of artificial intelligence that aims to enhance reader-expert calibration using a Web-based interactive scoring interface [11]. This provides continuous visual real-time feedback to readers using a color-coding scheme regarding concordance/discordance of their scores for MRI lesions with expert reader scores that are embedded in the scoring platform.

In this endeavor, we aimed to assess (1) the utility of an adult RETIC module for raters knowledgeable with the scoring of the SIS and SSS and (2) reliability of change scores for the SIS and SSS components.

## Methods

The protocol for this study was reviewed and approved by the Children’s Hospital of Philadelphia’s Committee for the Protection of Human Subjects (IRB 18-014988). A waiver of consent/parental permission, a waiver of assent, and a waiver of HIPAA authorization were granted for this retrospective study.

### Patients

We assessed MRI scans of children with spondyloarthritis (fulfilling the European Spondyloarthritis Study Group (ESSG), Assessment in Spondyloarthritis International Society (ASAS) criteria for spondyloarthritis, or International League of Associations for Rheumatology (ILAR) criteria for enthesitis-related arthritis (ERA) from two large tertiary care centers (University of California, San Francisco Benioff Children’s Hospital and the Children’s Hospital of Philadelphia) who underwent at least 2 pelvic MRI scans spaced at least 12 weeks apart and 2 years apart for assessment of the SIS and SSS change scores, respectively. Studies were a convenience sample and obtained at the treating physician’s discretion.

### Imaging

MRI studies included semicoronal T1-weighted and fluid-sensitive sequences. The SIS quantifies the presence, depth, and intensity of bone marrow edema (BME) on short tau inversion recovery (STIR) MRI (total score 0–72). The SSS quantifies the magnitude of fat metaplasia, erosion, backfill, ankylosis, and sclerosis using T1-weighted sequences (score 0–20 or 0–40 for each). Scoring for the SIS and SSS is done using six and five consecutive slices, respectively, through the cartilaginous part of the joint. Total scores for MRI studies with more than six slices scored for the SIS were calculated using the six consecutive slices with the highest score. Studies with fewer than six anatomically available slices as determined by at least half of the raters were evaluated using the maximum number of scored slices.

Digital Imaging and Communications in Medicine (DICOM)-based anonymized cases were scored in randomized order through the Canadian Research and Education (CaRE) Arthritis platform (CaREArthritis.com). Six raters (1 adult radiologist, 3 pediatric radiologists, 1 adult rheumatologist, 1 pediatric rheumatologist), knowledgeable with the scoring of the SIS and SSS [4, 8], scored studies blinded to clinical details and order. Two of the raters (WPM and RGL) developed the SPARCC SIS and SSS. All raters were first instructed to review a PowerPoint module that describes details of the methodology for scoring the SIJ using these methods with examples of pediatric images of MRI lesions in the SIJ. These PowerPoint slides also contain links to the DICOM series of pediatric images for each case so that the entire SIJ can be evaluated by scrolling through consecutive semicoronal slices through the SIJ.

After scoring exercise 1, all raters participated in the RETIC calibration module for both the SIS and SSS comprised of 20 adult cases consisting of 2 studies each. The two calibration modules provide continuous visual real-time feedback regarding concordance/discordance of reader scoring per SIJ quadrant according to benchmark scores from the SPARCC developers using a color-coding scheme. This real-time iterative feedback is conducted on a Web-based platform (available at CaREArthritis.com) that includes a data-entry scoring schematic directly on the DICOM image. Both modules have been validated for their capacity to enhance reliable detection of MRI lesions in the SIJ [12]. The study was scored in pairs blinded to timepoint. For the first 10 cases, raters received instantaneous feedback on concordance/discordance of scoring (inflammation, backfill, fat metaplasia, erosion, ankylosis; sclerosis not scored) after scoring each individual semicoronal slice. For the second 10 cases, feedback was provided after scoring the entire case. Intraclass correlation coefficient (ICC) with expert reader scores, including change scores, was provided after the first 10 cases, then again after the next 10 cases. A priori ICC acceptability thresholds for this calibration exercise were > 0.7 for SIS and > 0.5 for all SSS change scores [11]. After the interactive training module, we conducted a second scoring exercise. Exercises 1 and 2 were separated by 6 months.

### Analysis

Interobserver reliability of change scores was assessed using ICCs by rater background groups (all, pediatric radiologists, and SPARCC developers) and for all possible rater pairs using two-way random-effects models with absolute agreement and type single rater. Pre-specified acceptability thresholds for change scores were based upon standards listed on the CaREarthritis platform and acceptability thresholds used in prior published validation studies of the SPARCC SIS and SSS [1, 2]. Reliability for change scores is typically lower than for status scores when rating adult studies [11]. Hence, the lower pre-specified cut-offs than for status scores. Moreover, reliability for structural lesions is expected to be lower than for active lesions due to the smaller degree of change in structural lesions and hence greater difficulty in their reliable detection. In order to assess sensitivity to change, the standardized response mean (SRM) was calculated for the SIS and each of the SSS components. An SRM of < 0.2 was considered a trivial effect, 0.2–0.5 small effect, 0.5–0.8 moderate effect, and > 0.8 large effect [13]. All analyses were performed using Stata Statistical Software version 14.2 (StataCorp. 2015. Release 14. College Station, TX: StataCorp LP).

## Results

### Patients

Reading exercises 1 and 2 had a total of 30 unique subjects—26 in round 1 and 19 in round 2, with subject overlap between exercises. Each subject had 2 imaging studies performed at least 12 weeks apart for the SIS analyses. For the SSS analyses, imaging studies were separated by at least 2 years, reducing the eligible subject count to 12 and 18 for exercises 1 and 2, respectively. The median age of all evaluated subjects at baseline imaging was 13.3 years (IQR 10.2–15.6). Approximately half of the patients were male (60%) and the majority were HLA-B27-positive (63%). The median time between images included in the SIS analyses was 27.3 months (14.7–43.4), and SSS analyses were 36.4 months (IQR 27.4–53.7). Twenty-six (87%) subjects received biologic therapy at some point between imaging studies. Two imaging studies had at least half of the raters score fewer than six slices for the SIS. Table 1 shows the number of non-zero change scores reported by raters across all subjects for exercises 1 and 2.

**Table 1:** Absolute, non-zero (SSS), and ≥ 2 (SIS) change score findings for all raters

	***N***	% Non-zero	Median (IQR)	Min	Max
**Scoring exercise 1**					
Inflammation	113	72.4	14 (7–24)	1	53
Backfill	20	27.8	2 (1–3)	1	6
Fat Metaplasia	19	26.4	3 (1–7)	1	10
Sclerosis	29	40.3	4 (2–6)	1	15
Erosion	49	68.1	3 (2–8)	1	17
Ankylosis	4	5.6	2 (1.5–3.5)	1	5
**Scoring exercise 2**					
Inflammation	92	80.7	14.5 (4.5–29)	1	68
Backfill	42	38.9	3.5 (2–6)	1	16
Fat metaplasia	25	23.1	5 (3–10)	1	33
Sclerosis	47	43.5	4 (2–6)	1	10
Erosion	85	78.7	4 (2–7)	1	24
Ankylosis	13	12.0	5 (2–18)	1	20

Legend: *SIS* sacroiliac joint inflammation score, *SSS* sacroiliac joint structural score, *IQR* interquartile range

### Calibration activity

Calibration with the RETIC module was performed by all raters less than 2 weeks before the second scoring exercise (mean 5.2 days (range 0–14) for the SIS and 4.8 days (range 0–13) for the SSS).

### Reliability of change scores

**Fig. 1:**
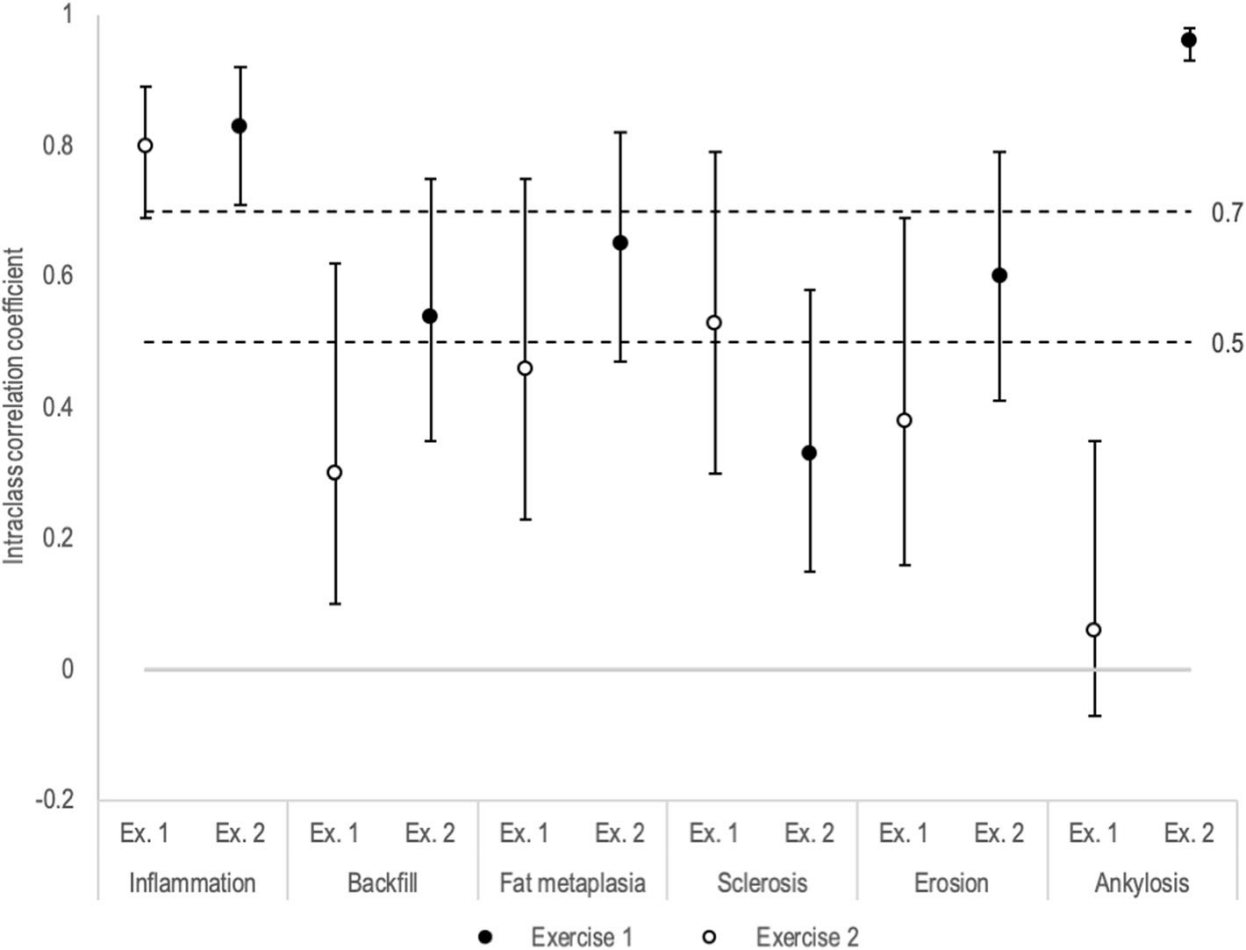
Change score intraclass correlation coefficients for all raters pre- and post-recalibration. Legend: SIS measures inflammation and had *N* = 26 and *N* = 19 subjects for scoring exercises 1 and 2. SSS components include backfill, fat metaplasia, sclerosis, erosion, and ankylosis, with *N* = 12 and *N* = 18 subjects for scoring exercises 1 and 2, respectively. Dotted horizontal lines represent the a priori acceptability thresholds for change scores and were ICC > 0.7 for SIS and > 0.5 for all SSS components. Solid error bars indicate the 95% confidence intervals. SIS sacroiliac joint inflammation score, SSS sacroiliac joint structural score, ICC intraclass correlation coefficient

Interobserver reliability for change scores from all raters for both scoring exercises are shown in Table 2. Figure 1 shows the difference in change score ICC before and after calibration with the most substantial improvement demonstrated in the ICCs for backfill, erosion, and ankylosis. After recalibration in scoring exercise 2, ICC for change scores was acceptable for the SIS (0.86, 95% CI 0.75–0.94) and all SSS components except sclerosis.

**Table 2:** Reliability of change scores for the SIS and SSS components

	Change scores ICC (95% CI)		
Component	All raters ***N*** = 6	Pediatric radiologists ***N*** = 3	SPARCC developers ***N*** = 2
**Scoring exercise 1**			
Inflammation	0.8 (0.69–0.89)	0.84 (0.7–0.92)	0.96 (0.9–0.98)
Backfill	0.3 (0.1–0.62)	0.49 (0.14–0.79)	− 0.74 (− 0.92–0.09)
Fat metaplasia	0.46 (0.23–0.75)	0.55 (0.19–0.82)	0.29 (− 0.34–0.73)
Sclerosis	0.53 (0.3–0.79)	0.49 (0.14–0.8)	0.46 (− 0.12–0.81)
Erosion	0.38 (0.16–0.69)	0.6 (0.26–0.85)	0.33 (− 0.19–0.74)
Ankylosis	0.06 (− 0.07–0.35)	0 (− 0.29–0.44)	0 (0–0)
**Scoring exercise 2**			
Inflammation	0.83 (0.71–0.92)	0.85 (0.7–0.93)	0.82 (0.6–0.93)
Backfill	0.54 (0.35–0.75)	0.52 (0.24–0.76)	0.88 (0.71–0.95)
Fat metaplasia	0.65 (0.47–0.82)	0.92 (0.84–0.97)	0.17 (− 0.3–0.58)
Sclerosis	0.33 (0.15–0.58)	0.38 (0.1–0.67)	0.41 (− 0.07–0.73)
Erosion	0.60 (0.41–0.79)	0.65 (0.4–0.84)	0.72 (0.4–0.89)
Ankylosis	0.96 (0.93–0.98)	0.94 (0.87–0.97)	1 (1–1)

Legend: SIS measures inflammation and had *N* = 26 and *N* = 19 subjects for scoring exercises 1 and 2. SSS components include backfill, fat metaplasia, sclerosis, erosion, and ankylosis with *N* = 12 and *N* = 18 subjects for scoring exercises 1 and 2, respectively. The acceptability thresholds for change scores were ICC > 0.7 for SIS and > 0.5 for all SSS components

*SIS* sacroiliac joint inflammation score, *SSS* sacroiliac joint structural score, *ICC* intraclass correlation coefficient

ICC for change scores calculated per reader pair for scoring exercise 2 are summarized in Table 3. The mean per reader pair SIS ICC was 0.83 and 93% of pairs achieved the pre-specified acceptability cut-off of > 0.7. All SSS components except sclerosis achieved a mean per reader pair ICC for change score > 0.5. The percentage of reader pairs achieving an ICC > 0.5 ranged from 27 to 100% across all components: backfill (53%), fat metaplasia (47%), sclerosis (27%), erosion (67%), and ankylosis (100%). There was variability in agreement among the various reader pairs, but there was no consistent pair for the lowest or highest ICC. There were no rater pairs with the lowest ICC for more than two components. Confirmatory analysis of the reliability findings was carried out using Krippendorff’s alpha and nearly identical results were achieved.

**Table 3:** Summary of change score ICC for all rater pairs

Component	All rater pairs Mean (range)	Rater pairs with ICC > 0.50 *N* (%)	Rater pairs with ICC > 0.70 *N* (%)
Inflammation	0.83 (0.68–0.95)	15 (100%)	14 (93.3%)
Backfill	0.5 (0.16–0.88)	8 (53.3%)	3 (20%)
Fat metaplasia	0.57 (0.09–0.99)	7 (46.7%)	7 (46.7%)
Sclerosis	0.36 (0.13–0.68)	4 (26.7%)	0 (0%)
Erosion	0.58 (0.32–0.77)	10 (66.7%)	4 (26.7%)
Ankylosis	0.96 (0.9–1)	15 (100%)	15 (100%)

Legend: *ICC* intraclass correlation coefficient

### Sensitivity to change

**Fig. 2:**
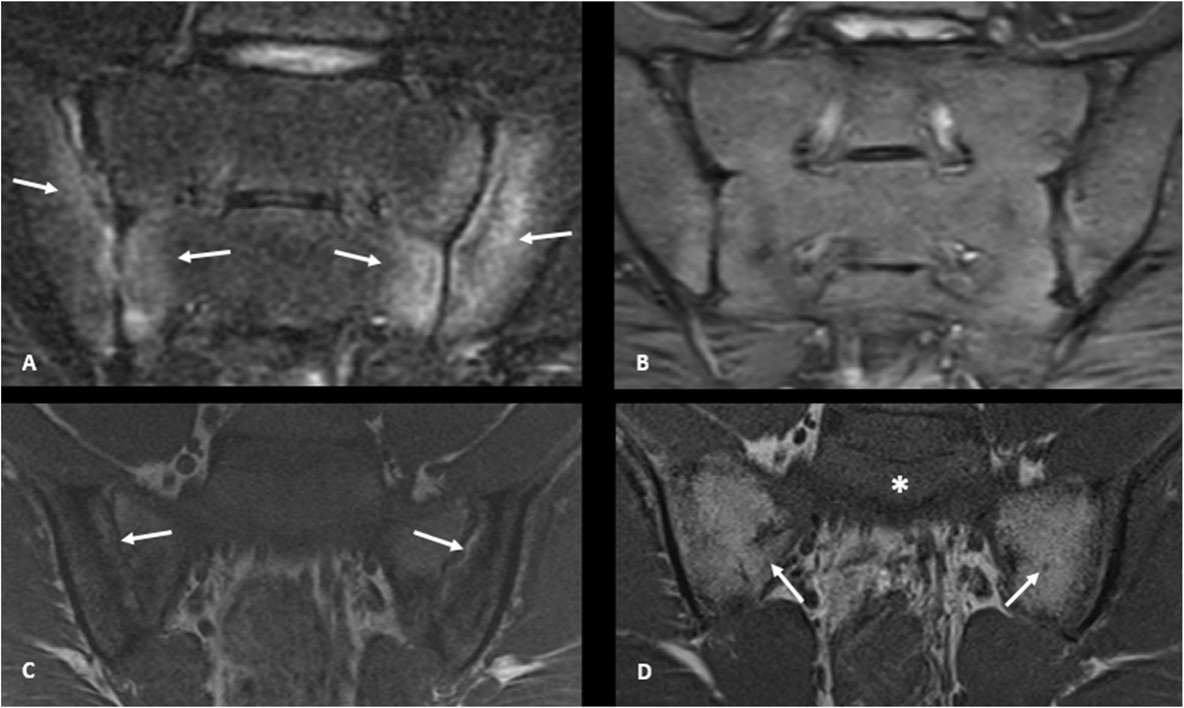
Interval change in SIS and SSS lesions in children. Legend: Coronal oblique STIR images of the sacroiliac joints in a 12-year-old girl (**a**) and at age 16 years (**b**). **a** There is marrow edema along both the iliac and sacral aspects of the sacroiliac joints (arrows), left greater than right. Findings are consistent with active sacroiliitis. On follow-up imaging 4 years later (**b**), the marrow edema has resolved. Coronal oblique T1-weighted images of the sacroiliac joints in a 15-year-old boy (**c**) and at age 19 years (**d**). **c** There are erosions along both aspects of the iliac bones. In addition, there is high T1-weighted signal within both joints consistent with backfill (arrows). On follow-up imaging 4 years later (**d**), there is ankylosis of both joints with fat metaplasia, as demonstrated by fatty marrow signal along the fused sacroiliac joints (arrows). Note the normal appearance of the marrow within the upper sacrum demonstrated low signal (*)

In order to assess sensitivity to change, we calculated the SRM for the SIS and each SSS component (Table 4). SRM for the SIS (0.90) and SSS erosion (0.96) were large. Backfill (0.55) and sclerosis (0.70) demonstrated moderate sensitivity and fat metaplasia (0.36) and ankylosis (0.28) had small effect. A sub-analysis using the SPARCC developer scores demonstrated similar results (Table 4). Figure 2 shows two cases where substantial change was noted between images in the SIS and several SSS lesions.

**Table 4:** Standardized response means (SRM) for SIS and SSS components

Component	All raters Mean (range)	SPARCC developers
Inflammation	0.93 (0.9–0.95)	0.91
Backfill	0.55 (0.35–0.96)	0.38
Fat metaplasia	0.36 (0.34–0.47)	0.30
Sclerosis	0.70 (0.57–0.88)	0.67
Erosion	0.96 (0.7–1.39)	0.78
Ankylosis	0.28 (0.24–0.31)	0.25

Legend: An SRM of < 0.2 was considered a trivial effect, 0.2–0.5 small effect, 0.5–0.8 moderate effect, and > 0.8 large effect [12]

*SIS* sacroiliac joint inflammation score, *SSS* sacroiliac joint structural score

## Discussion

Our results demonstrate that RETIC improved the reliability of the SPARCC SIS and SSS change scores, even for raters familiar with these methods who had participated in previous scoring exercises. Although most confidence intervals overlapped between the two scoring exercises, there was a notable and consistent trend of improvement in the ICCs with calibration and narrowing of the confidence intervals. We also demonstrated the SPARCC SIS and all SSS components, except sclerosis, are reliable measures to quantify change over time in children. The reliability thresholds for change scores, ICC > 0.7 for SIS and > 0.5 for all SSS components, were based upon standards listed on the CaREarthritis platform and acceptability thresholds used in prior published validation studies of the SPARCC SIS and SSS [1, 2]. These pre-specified cut-offs reflect that change score reliability was expected to be lower than the status scores based on prior studies [11]. Further, the reliability for the SSS lesions was anticipated to be lower than for SIS lesions due to the smaller degree of change in structural lesions and hence greater difficulty in their reliable detection.

The change scores from scoring exercise 1 demonstrate the necessity of such calibration for our group, especially for those who do not routinely read pediatric studies or score the SSS and SIS. Even the SPARCC developers had improvement in pairwise ICC change scores in 3 of 5 SSS components after RETIC. Sclerosis was the component for which the change score ICC did not meet the preset acceptability threshold after calibration. A couple of factors may contribute to this finding. First, the scoring of sclerosis is not included in the RETIC module since sclerosis is not part of the SSS scoring method in adults. Second, the reliability for change scores is typically lower than for status scores when rating adult studies [11], hence the pre-specified cut-offs of > 0.7 and > 0.5 for the SIS and SSS components, respectively. Third, scoring of change in structural lesions is less reliable as estimated using the ICC due to the much slower evolution of structural lesions.

Interactive Web-based calibration of the SIS and SSS using DICOM images, game theory, and real-time iterative feedback for scoring of adult studies is both feasible and effective [11]. RETIC using the same technology through the CaREarthritis.com platform and 20 adult cases improved the reliability of change scores for all raters, regardless of background expertise. The value of RETIC was diminished among experienced raters for the SIS who achieved high levels of agreement for change scores pre-calibration—pediatric radiologist agreement remained almost exactly the same and SPARCC developer agreement decreased between the exercises pre- and post-calibration. The same experienced rater groups saw a largely positive effect on change scores across SSS components. These results highlight that raters of all levels can benefit from SSS recalibration whereas SIS recalibration may not be required on the same frequency schedule for raters experienced with identifying bone marrow signal.

The main drawback of using this particular calibration system in a pediatric study is the reliance on adult studies. For the purpose of investigating the viability of the SPARCC scoring in children over time, using the available calibration tools was considered most prudent until we gathered evidence supporting the use of these measures in pediatrics. Given age-related differences in the appearance of the sacroiliac joint, a pediatric-based refresher module would be ideal and should include the full array of SSS components used in children, including sclerosis. We suspect generating pediatric-specific tools will only strengthen the reliability of the SPARCC SIS and SSS. Future calibration exercises could also be designed to terminate the calibration exercise once an acceptable ICC is attained for all components versus compelled completion of all 20 cases. The optimal lapse in time after which recalibration should be performed remains unclear but could be evaluated in an iterative fashion. These promising results suggest pursuing a pediatric-specific calibration system would be worthwhile.

The SPARCC SIS change scores had very good reliability (≥ 0.8 but < 0.9) using both an overall and per reader pair ICC analysis, even before calibration. All SPARCC SSS components except sclerosis met the acceptability threshold for overall and pairwise change score ICCs. The by-rater pair analysis for the SIS and SSS components demonstrated significant variability in the agreement among various rater pairs, which is not surprising given the varying backgrounds and expertise of the 6 raters. However, there was no rater pair that was a consistent outlier for the lowest or highest ICC. The SIS and SSS erosion demonstrated large sensitivity to change while backfill and sclerosis demonstrated moderate sensitivity to change.

## Conclusion

In conclusion, we demonstrated the necessity and effectiveness of calibration prior to SIS and SSS scoring exercises as well as the reliability of the SIS and SSS component change scores (except sclerosis). Future work is needed to optimize the calibration toolset for pediatric studies.

## Data Availability

The datasets used and/or analyzed during the current study are available from the corresponding author on reasonable request.

## Abbreviations

ASAS: Assessment of Spondyloarthritis International Society
BME: Bone marrow edema
CaRE: Canadian Research and Education
DICOM: Digital Imaging and Communications in Medicine
ERA: Enthesitis-related arthritis
ESSG: European Spondyloarthritis Study Group
ICC: Intraclass correlation coefficient
ILAR: International League of Associations for Rheumatology
MRI: Magnetic resonance imaging
RETIC: Real-time iterative calibration
SIJ: Sacroiliac joint
SIS: Sacroiliac joint inflammation score
SPARCC: Spondyloarthritis Research Consortium of Canada
SRM: Standardized response mean
SSS: Sacroiliac joint structural score
STIR: Short tau inversion recovery
